# Efficacy and safety of thiazolidinediones in diabetes patients with renal impairment: a systematic review and meta-analysis

**DOI:** 10.1038/s41598-017-01965-0

**Published:** 2017-05-11

**Authors:** Wen Wang, Xu Zhou, Joey S. W. Kwong, Ling Li, Youping Li, Xin Sun

**Affiliations:** 1 Chinese Evidence-based Medicine Center, West China Hospital, Sichuan University, Chengdu, 610041 China; 2 Clinical Research and Evaluation Unit, West China Hospital, Sichuan University, Chengdu, China; 3 Center for Evidence-Based Medicine and Clinical Research, Taihe Hospital, Hubei University of Medicine, Shiyan, Hubei China; 4 Research Center of Evidence-based Medicine, School of Basic Medicine, Jiangxi University of Traditional Chinese Medicine, Nanchang, Jiangxi China

## Abstract

We conducted a systematic review and meta-analysis to evaluate the efficacy and safety of TZDs in treatment of diabetes mellitus patients with renal impairment. We searched PubMed, EMBASE and Cochrane Central Register of Controlled Trials. Randomized controlled trials (RCTs), cohort studies, and case-control studies that investigated the effects of TZDs in patients with diabetes and renal impairment were eligible. Outcomes included glycosylated hemoglobin, fasting plasma glucose, serum lipids, and patient-important outcomes (i.e. hypoglycemia, weight, edema, cardiovascular events and mortality). 19 RCTs and 3 cohort studies involving 21,803 patients with diabetes and renal impairment were included. Meta-analysis of RCTs showed that TZDs could significantly reduce HbA1c (MD −0.64, 95%CI −0.93 to −0.35), FPG (MD −26.27, 95%CI −44.90 to −7.64) and increase HDL levels (MD 3.70, 95%CI 1.10, 6.29). TZDs could increase weight (MD 3.23, 95% CI 2.29 to 4.16) and risk of edema (RR 2.96, 95% CI 1.22 to 7.20). Their effects on risk of hypoglycemia (RR 1.46, 95% CI 0.65 to 3.29), heart failure (RR 0.64, 95% CI 0.15 to 2.66), angina (RR 1.45, 95% CI 0.23 to 8.95) and all-cause mortality (RR 0.40, 95% CI 0.08 to 2.01) are uncertain. Results from cohort studies were similar to RCTs.

## Introduction

The prevalence of diabetes mellitus continues to rise worldwide^1^. Chronic kidney disease, a common complication in diabetes patients, has recently become the leading cause of end-stage renal disease (ESRD) requiring dialysis in most countries^2^.

Treatment options for diabetic patients with chronic kidney disease is limited, especially in patients with ESRD. With their deteriorated renal function, many oral hypoglycemic drugs (e.g. metformin) are not recommended for patients with severe chronic kidney disease^3^. The thiazolidinediones (TZDs) (rosiglitazone and pioglitazone) are activated receptor gamma (PPAR-γ) antidiabetic agents, and are mainly metabolized by liver. They do not require dose adjustment in patients with renal impairment^4, 5^, and may have renal protective effects. A meta-analysis indicated that treatment with TZDs significantly decreased urinary albumin and protein excretion in patients with diabetes^6^. In addition to renal benefits, pioglitazone has been shown to improve a number of intermediate markers of cardiovascular diseases, such as blood pressure and serum lipids^7^.

However, cardiovascular safety of TZDs in patients with diabetes mellitus patients has become a matter of major controversy, especially for rosiglitazone. Several meta-analyses showed that the risk of myocardial infarction (MI) and heart failure was significantly increased by rosiglitazone^8, 9^. In 2007, US Food and Drug Administration (FDA) restricted treatment of rosiglitazone only in new patients who are unable to achieve glucose control with other drugs or unable to take pioglitazone, and current users who are benefiting from this drug and choose to continue using it^10^. The Rosiglitazone Evaluated for Cardiac Outcomes and Regulation of glycaemia in Diabetes (RECORD) study, however, did not rule out an elevated risk of myocardial infarction amongst participants treated with rosiglitazone^11^. The Veterans Affairs Diabetes Trial (VADT) even found that use of rosiglitazone was associated with decreased risk of cardiovascular composite outcome and cardiovascular death^12^. Recently FDA repealed restriction of rosiglitazone. Though accumulating studies focused on cardiovascular safety of TZDs treatment, most of these studies excluded patients with obvious renal impairment. The safety of TZDs in treatment of diabetes patients with renal impairment has still been uncertain. Considering high prevalence of cardiovascular events in patients with renal impairment, whether TZDs increase the risk of heart failure, myocardial infraction and mortality has been a major concern of clinician.

Most of reported studies of TZDs treating in diabetes patient with renal impairment were small sample sizes (especially in randomized control trials) and had conflicting findings on cardiovascular outcomes^13–17^. A cohort study found that TZDs use was associated with better survival in hemodialysis patients with type 2 diabetes^14^, but another cohort study found that diabetes patients prescribed rosiglitazone had significantly higher all-cause mortality and cardiovascular mortality^15^. Except for mortality, whether treatment of TZDs in diabetes patients with renal impairment increase the risk of heart failure was inconsistent^16, 17^. Though guideline approved treatment of TZDs in patients with chronic renal failure^2, 18^, but these recommends mainly based on pharmacokinetics not clinical researches.

Consequently, we conducted this systematic review and meta-analysis to investigate the efficacy and safety of TZDs in treatment of patients with diabetes mellitus and renal impairment.

## Results

**Figure 1:**
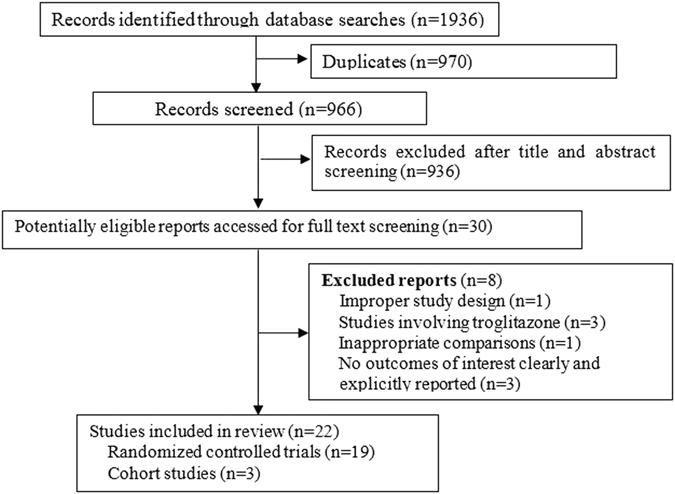
Flow chart of article selection.

We identified a total 1,936 potentially relevant reports in the initial retrieval. Finally, 22 studies were included in data analysis, including 19 RCTs (n = 1,818) and 3 cohort studies (n = 19,985) (Fig. 1).

### Study characteristics

Table 1 summarized the characteristics of the 22 included studies. The 19 RCTs involved a total of 1,818 participants, with mean age ranging from 43.4 to 71.1 years, mean baseline HbA1c 6.9 to 9.2%, mean fasting plasma glucose 135.7 to 205.2 mg/dl, and mean duration of diabetes 5.5 to 17.5 years. Of the 19 RCTs, one (5.3%) enrolled patients undergo renal transplantation, five (26.3%) enrolled dialysis patients, and thirteen (68.4%) trials included patients with mild to moderate renal impairment. Fourteen (73.7%) trials used pioglitazone as intervention, four (21.1%) used rosiglitazone, one (5.3%) used both pioglitazone and rosiglitazone.

**Table 1:** Characteristics of included studies.

Author	Intervention	Drug treatments used across groups	No. of patients	Male patients (No, %)	Age (years)	Duration of diabetes (years)	FPG (mg/dl	HbA1c (%)	Type of kidney impairment	Follow-up (week)	Follow-up rate
**Randomized controlled trials**											
Abe^19^	I: pioglitazone	voglibose	16	9 (56.3)	70.1 (20.4)	NR	193 (91.2)	7.05 (0.6)	ESRD (hemodialysis)	12	100%
	C: no additional drugs		15	9 (60)	65.6 (10.8)	NR	178.1 (49.2)	7.29 (1.2)			
Abe^20^	I: pioglitazone	oral antidiabetic agents	20	12 (60)	71.1 (31.8)	NR	189.2 (100.2)	6.94 (0.7)	ESRD (hemodialysis)	24	100%
	C: no additional drugs		20	12 (60)	68.8 (25.9)	NR	180.1 (74.7)	7.11 (1.4)			
Abe^16^	I: pioglitazone	oral antidiabetic agents	31	21 (67.7)	65.2 (12.1)	16.6 (5.5)	139 (45)	7.4 (0.5)	ESRD (hemodialysis)	96	96.80%
	C: no additional drugs		32	22 (68.8)	67.2 (9.4)	16.3 (5.5)	138 (40)	7.4 (0.5)			
Agarwal^17^	I: pioglitazone	other oral antidiabetic agents or insulin	22	22 (100)	67 (8.5)	15.9 (8.0)	147 (58)	7.7 (2.2)	non-ESRD (urine protein/creatinine ratio of >1.0 g/g)	16	90.10%
	C:glipizide		22	22 (100)	64.1 (8.4)	14.3 (9.8)	155 (79)	7.7 (2.5)			
Agrawal^21^	I: rosiglitazone	sulfonylureas	145	75 (51.7)	65.8 (7.0)	10.2 (7.0)	201.6 (48.6)	9.2 (1.3)	non-ESRD (mild to moderate RI)	24	96.00%
	C:placebo		156	85 (54.5)	67.0 (7.0)	11.6 (8.0)	205.2 (48.6)	9.0 (1.4)			
Arashnia^22^	I: pioglitazone	insulin	29	20 (69.0%)	44.4 (14.2)	NR	137.4 (28.8)	NR	ESRD (renal transplantation)	16	100%
	C:placebo		29	18 (62.1%)	43.4 (13.7)	NR	141.7 (67.6)	NR			
Banerji^23^	I: TZDs	metformin	231	119 (51.5)	61.1 (8.3)	5.8 (4.8)	162.1 (34.2)	7.8 (0.7)	no-ESRD (mild RI)	12	91.40%
	C:vildagliptin		464	241 (51.9)	61.3 (8.5)	5.5 (5.4)	163.8 (39.6)	7.8 (0.8)			
Chan^24^	I: rosiglitazone	previous antidiabetic medication	35	24 (68.6)	62 (10)	NR	97.2 (21.6)	NR	non-ESRD (CKD Stages 3–4)	8	98.60%
	C:placebo		35	26 (74.3)	62 (10)	NR	99 (21.6)	NR			
Galle^25^	I: pioglitazone	insulin	20	14 (70)	68.9 (6.8)	13.8 (9.8)	152.5 (45.0)	7.4 (0.9)	ESRD (hemodialysis)	24	66.70%
	C:placebo		19	13 (68.4)	69.6 (9.4)	12.4 (8.2)	156.6 (43.6)	7.7 (0.9)			
Jin^26^	I: pioglitazone	insulin	30	16 (53.3)	52.8 (12.3)	NR	NR	NR	non-ESRD (CKD Stages 3–4)	52	100%
	C: no additional drugs		30	16 (53.3)	51.1 (11.2)	NR	NR	NR			
Katavetin^27^	I: pioglitazone	other oral antidiabetic agents and/or insulin	24	6 (25)	61.4 (10.3)	13.6 (6.9)	137.3 (114.7–164.4)	8.6 (2)	non-ESRD (Proteinuria > 500 mg/day)	12	NR
	C:placebo		16	8 (50)	62.3 (10.4)	13.8 (7.2)	135.7 (115.3–159.7)	8.3 (2)			
Morikawa^28^	I: pioglitazone	other oral antidiabetic agents or insulin	36	27 (75.0)	62.5 (10.2)	9.5 (1)	NR	7.9 (1.2)	non-ESRD (UACR 30–300 mg/g Cr)	52	90%
	C:metformin		32	22 (67.7)	62.4 (8.4)	11.6 (13)	NR	8 (1.1)			
Nakamura^32^	I: pioglitazone	none	15	7 (46.7)	60 (13)	16 (4)	NR	NR	non-ESRD (UAE 20–200 ug/ml)	12	NR
	C:glbenclamide		15	8 (53.3)	61 (10)	14 (4)	NR	NR			
Nakamura^31^	I: pioglitazone	diet and/or glibenclamide	14	18*	52.5 (10.2)*	NR	186 (24)	8.4 (1.3)	non-ESRD (UAE 20–200 ug/ml)	24	NR
	C:placebo		14			NR	176 (22)	8.0 (1.0)			
Nakamura^29^	I: pioglitazone	none	15	9 (60)	56.5 (12.0)	17.5 (4.5)	NR	7.9 (1.3)	non-ESRD (UAE 20–200 ug/ml)	52	100%
	C:glibenclamide		15	8 (53.3)	55.0 (11.5)	17.0 (4.8)	NR	7.8 (1.4)			
Nakamura^30^	I: pioglitazone	none	17	9 (52.9)	56.0 (13.0)	16.0 (5.0)	NR	8.0 (1.4)	non-ESRD (UAE 20–200 ug/ml)	52	100%
	C:glibenclamide		18	10 (55.6)	53.5 (12.0)	16.5 (5.5)	NR	7.8 (1.3)			
Pistrosch^33^	I: rosiglitazone	previous antidiabetic medication	14	12 (85.7)	65.4 (9.6)	NR	169.2 (57.6)	7.0 (3)	non-ESRD (GFR < 60 ml/min)	52	NR
	C:placebo		14	12 (85.7)	66.5 (8.5)	NR	142.2 (52.2)	7.3 (3)			
Wong^34^	I: rosiglitazone	insulin	26	NR	62.9 (7.3)	NR	NR	7.3 (1.3)	ESRD (peritoneal dialysis)	24	98.10%
	C: no additional drugs		26	NR	61.6 (9.7)	NR	NR	7.2 (1.3)			
Yanagawa^35^	I: pioglitazone	none	19	13 (68.4)	54.0 (10.3)	6.7 (5.2)	186.0 (30.0)	8.3 (0.7)	non-ESRD (UACR 30–300 mg/g)	12	100%
	C:gliclazide		21	15 (71.4)	54.0 (11.1)	6.0 (4.8)	167.0 (31.0)	8.3 (0.9)			
**Cohort studies**											
Brunelli^14^	I: TZDs	antidiabetic medication	353	187 (53.0)	64.4 (11.8)	NR	NR	NR	ESRD (hemodialysis)	39	NR
	C: no additional drugs		2832	1499 (52.9	64.0 (13.3)	NR	NR	NR			
Chen^36^	I: TZDs	oral antidiabetic agents or insulin	1224	596 (48.7)	65.1 (10.5)	6.4 (2.5)	NR	NR	ESRD (GFR < 15 mL/min)	24	NR
	C: no additional drugs		11126	5527 (49.7)	66.1 (11.6)	6.0 (3.2)	NR	NR			
Ramirez^15^	I: rosiglitazone	oral antidiabetic agents	177	82 (46.3)	63.7 (11.7)	NR	187.3 (99.3)	NR	ESRD (hemodialysis)	24	96.00%
	C:placebo		2050	1046 (51.0)	63.5 (12.3)	NR	171.4 (82.7)	NR			

Abbreviation: FPG = fasting plasma glucose, HbA1c = hemoglobin A1c, GFR = glomerular filtration rate, UACR = urinary albumin to creatinine ratio, UAE = urinary albumin excretion, ESRD = end-stage renal disease, NR = not reported.

Continuous variables are presented as mean (standard deviation) or median (interquartile range).

*Characteristics of total patients.

Of the three cohort studies, one was prospective and two were retrospective studies. These cohort studies included a total of 19,985 participants; mean age ranged from 63.5 to 66.1 years and mean follow-up 24 week to 270 days. All these cohort studies enrolled ESRD patients.

### Risk of bias assessment

All the 19 RCTs were at moderate to high risk of bias^16, 17, 19–35^ (Supplementary Table 1). Of the three cohort studies, one^15^ was at low risk of bias, one moderate risk^14^ and another^36^ high risk. (Supplementary Table 2).

### Publication bias assessment

Publication bias was investigated using the funnel plot and Egger’s tests. No evidence of publication bias was found for the outcomes of FPG, HbA1c, TC, TG, and HDL. Owing to the limited numbers of the included studies or low events rate, publication bias investigation was not performed for other outcomes.

### Efficacy of TZDs

#### HbA1c

**Figure 2:**
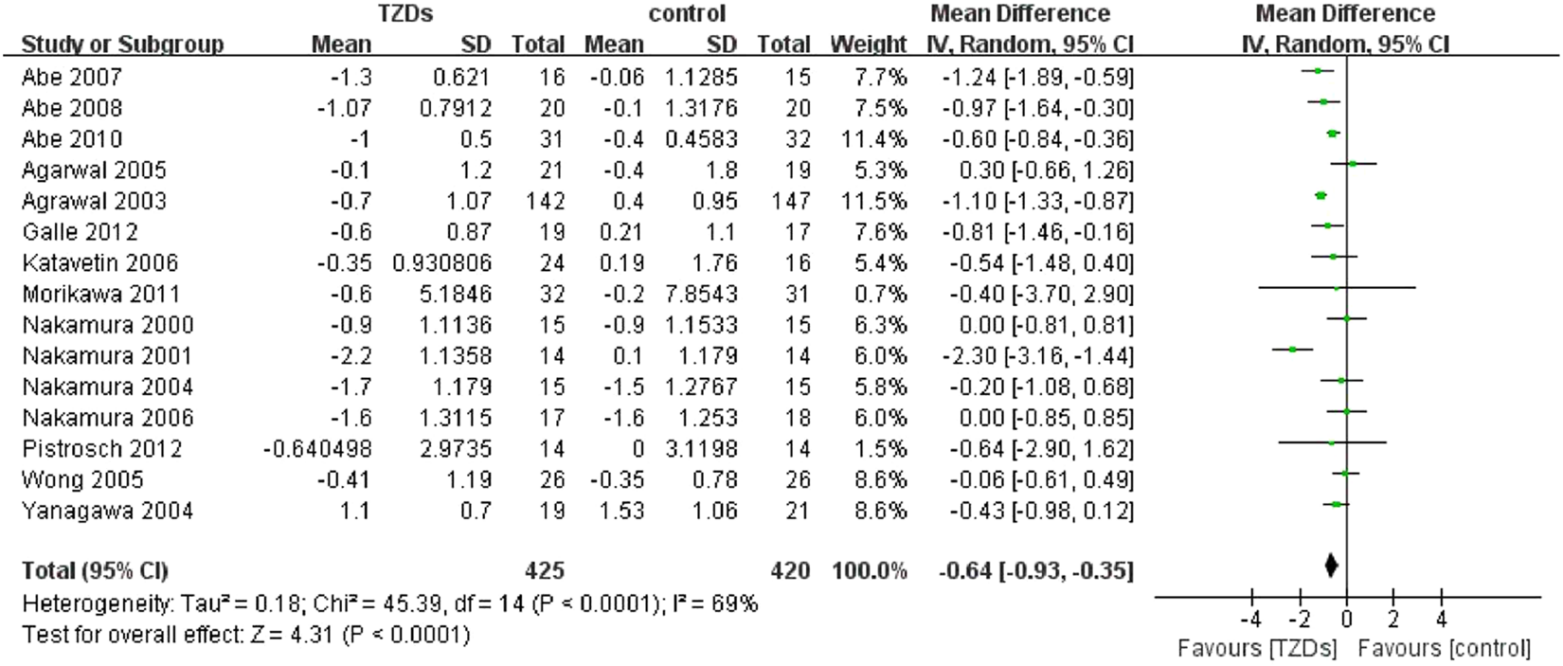
Change in HbA1c among patients with diabetes mellitus and renal impairment receiving TZDs versus control from RCTs.

Fifteen RCTs reported change of HbA1c from baseline. Compared with all controls, HbA1c in TZDs group significantly decreased, with substantial statistical heterogeneity (MD −0.64, 95% CI −0.93 to −0.35, I^2^ = 69%) (Fig. 2). Subgroup analysis showed that, compared with placebo or no additional drugs, levels of HbA1c in TZDs groups were significantly lower (MD −0.90, 95% CI −1.24 to −0.56, I^2^ = 73%), while no significance between groups was observed when compared with active drugs (MD −0.16, 95% CI −0.50 to 0.18, I^2^ = 0%; test for subgroup differences: P = 0.003) (Supplementary Table 3).

#### Fasting plasma glucose

**Figure 3:**
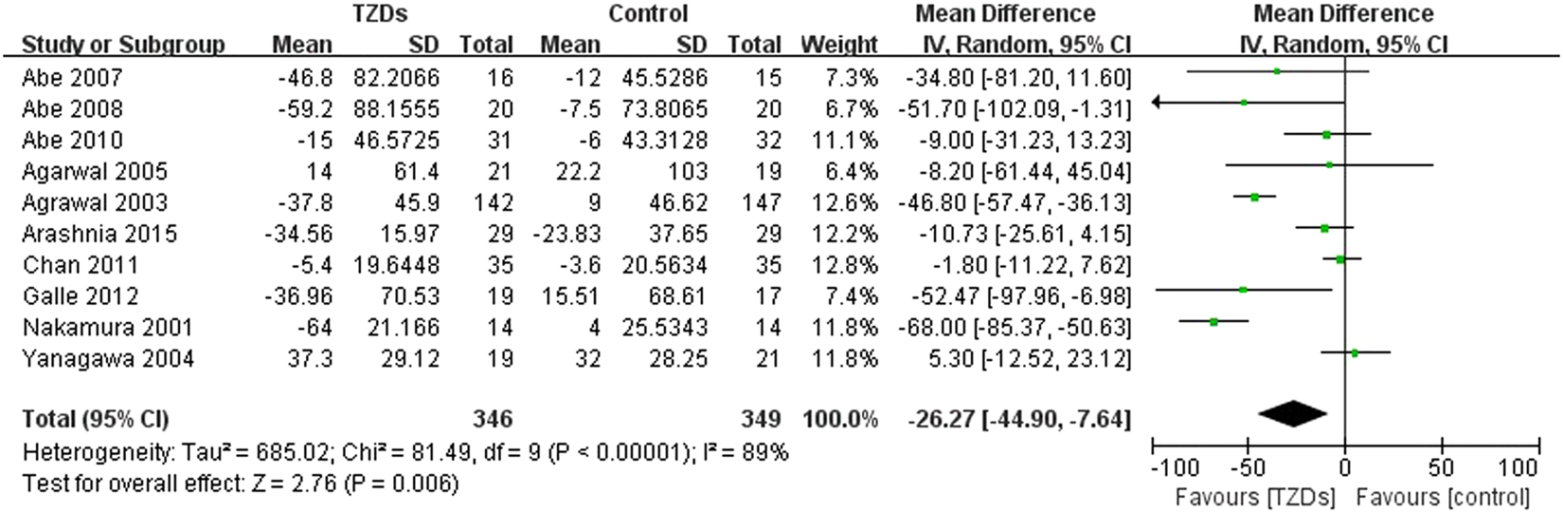
Change in FPG among patients with diabetes mellitus and renal impairment receiving TZDs versus control from RCTs.

Changes in FPG from baseline were reported in 10 RCTs. Compared with controls, treatment of TZDs was associated with a significant decrease in FPG levels, with considerable statistical heterogeneity (MD −26.27, 95% CI −44.90 to −7.64, I^2^ = 89%) (Fig. 3). Subgroup analysis showed that, when compared with placebo or no additional drugs, TZDs significantly decreased FPG levels (MD −32.26, 95% CI −53.13 to −11.39, I^2^ = 90%); comparison with active drugs as controls illustrated a lack of significant effect (MD 3.94, 95%CI −12.96 to 20.84, I^2^ = 0%; test for subgroup differences: P = 0.008) (Supplementary Table 3).

#### Serum lipids and lipoproteins

**Figure 4:**
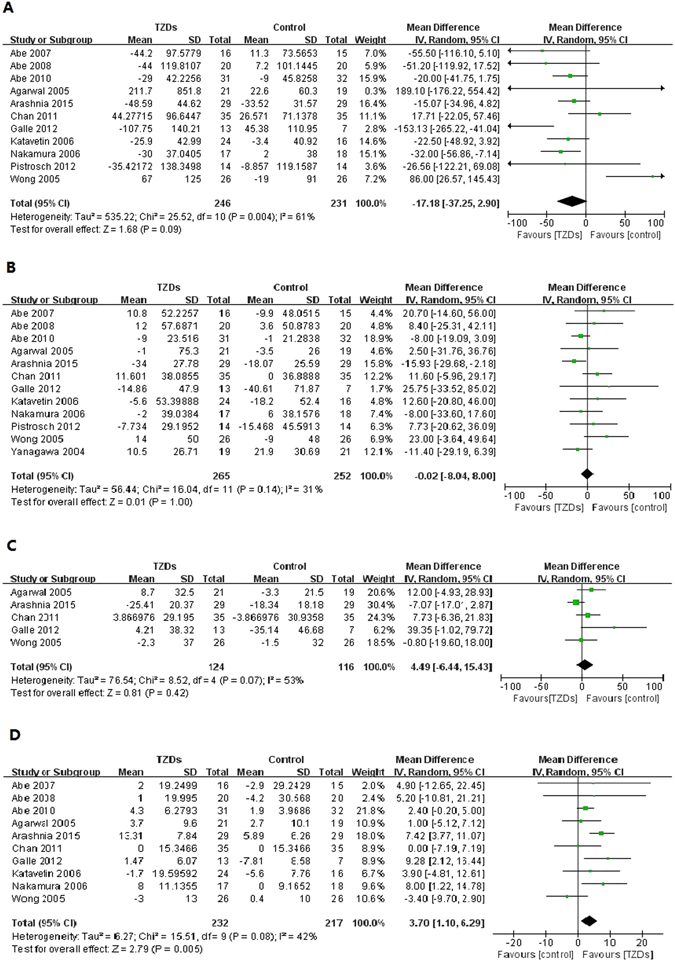
Changes in serum lipids and lipoproteins among patients with diabetes mellitus and renal impairment receiving TZDs versus control from RCTs. (**A**) Change in triacylglycerol (TG), (**B**) Change in total cholesterol (TC), (**C**) Change in low-density lipoprotein (LDL), (**D**) Change in high-density lipoprotein (HDL).

A total of 11 trials reported changes in triacylglycerol (TG). Compared with controls, TG in TZDs group had no significant decrease (MD −17.18, 95% CI −37.25 to 2.90, I^2^ = 61%) (Fig. 4A). Subgroup analysis showed that, compared with controls, levels of TG in pioglitazone group were significantly lower (MD −26.38, 95% CI −40.56 to −12.19, I^2^ = 25%); no significance was observed between rosiglitazone group and controls. (MD 31.81, 95% CI −24.73 to 88.35, I^2^ = 61%; test for subgroup differences: P = 0.05) (Supplementary Table 3).

Twelve trials reported changes in total cholesterol (TC), no significant differences between groups were observed in the analysis of TC (MD −0.02, 95% CI −8.04 to 8.00, I^2^ = 31%) (Fig. 4B). Subgroup analysis showed that, compared with controls, treatment of pioglitazone significantly decreased TC levels (MD −7.00, 95% CI −13.77 to −0.23, I^2^ = 0%), but treatment rosiglitazone significantly increased TC levels (MD 13.51, 95% CI 0.48 to 26.54, I^2^ = 0%; test for subgroup differences: P = 0.006) (Supplementary Table 3).

Five trials reported changes in low-density lipoprotein (LDL). There were no significant changes in LDL levels between TZDs group and controls (MD 4.49, 95% CI −6.44 to 15.43, I^2^ = 53%) (Fig. 4C). Subgroup analysis showed that, compared with controls, LDL levels had no significant decrease both in pioglitazone group (MD 8.30, 95% CI −12.82 to 29.41, I^2^ = 73%) and rosiglitazone group (MD 4.66, 95% CI −6.61 to 15.94, I^2^ = 0%; test for subgroup differences: P = 0.77) (Supplementary Table 3).

Ten trials reported changes in high-density lipoprotein (HDL). HDL in TZDs group was significant increase, and the heterogeneity was moderate (MD 3.70, 95% CI 1.10 to 6.29, I^2^ = 42%) (Fig. 4D). Subgroup analysis by type of TZDs showed that treatment of pioglitazone (MD 4.84, 95% CI 2.50 to 7.18, I^2^ = 22%), but not rosiglitazone (MD −1.92, 95% CI −6.66 to 2.82, I^2^ = 0%; test for subgroup differences: P = 0.01), significantly elevated HDL levels (Supplementary Table 3).

### Safety of TZDs

#### Hypoglycemia

Six trials involving 1,178 participants reported hypoglycemia, of which two trials^17, 23^ compared TZDs versus active drugs (sufonylureas, DPP-4), and four trials^16, 21, 25, 27^ compared TZDs versus placebo/no additional drugs. One trial^23^ used both rosiglitazone and pioglitazone as intervention, four trials^16, 17, 25, 27^ used pioglitazone and one trial^21^ used rosiglitazone as intervention. Meta-analysis suggested that, compared with controls, there was no significant differences between groups in the risk of hypoglycemia and the heterogeneity was low (RR 1.46, 95% CI 0.65 to 3.29, I^2^ = 0%) (Supplementary Fig. 1).

One cohort study^36^ involving 12,350 participants reported hypoglycemia. There was no significant difference in the risk of hypoglycemia between TZDs and control (RR 0.99, 95% CI 0.65 to 1.52).

#### Weight change

**Figure 5:**
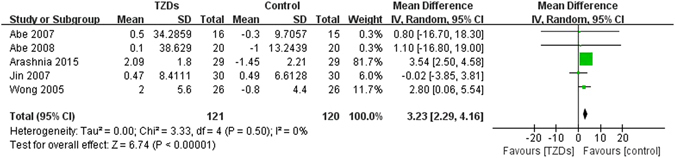
Change in weight among patients with diabetes mellitus and renal impairment receiving TZDs versus control from RCTs.

Five trials (n = 241) reported changes in weight, of which four trials enrolled patients with ESRD and treated patients with pioglitazone. All trials used placebo/no additional drugs as controls. Compared with control group, treatment of TZDs significantly increased body weight (MD 3.23, 95% CI 2.29 to 4.16, I^2^ = 0%) (Fig. 5). Of these five trails, two trials reported changes in dry weight. Considering there may be some differences between dry weight and total weight, we also did meta-analysis respectively. The results showed that total weight (MD 2.82, 95% CI 1.17 to 4.47, I^2^ = 38%), but not dry weight (MD 0.95, 95% CI −11.57 to 13.46, I^2^ = 0%) significantly increased in TZDs group. No cohort study reported weight changes.

#### Edema

**Figure 6:**
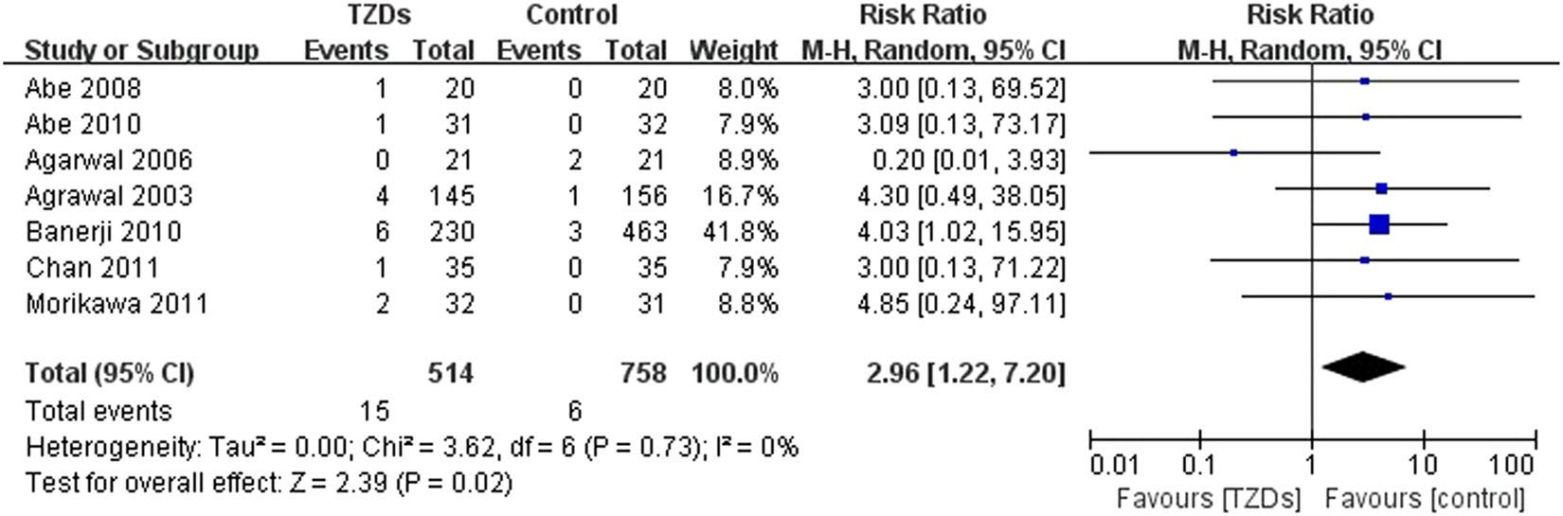
Risk of edema in patients with diabetes mellitus and renal impairment for the TZDs versus control groups from RCTs.

Seven trials reported edema or worsening edema, of which three trials^17, 23, 28^ compared TZDs versus active drugs, five trials^17, 21, 23, 24, 28^ enrolled patients with non-ESRD, and the other two trials^16, 20^ included patients with ESRD. One trial^23^ compared TZDs (both rosiglitazone and pioglitazone) versus control, four trials^16, 17, 20, 28^ compared pioglitazone versus control and the other two trials^21, 24^ compared rosiglitazone versus control. Meta-analysis of seven RCTs showed that the risk of edema significantly increased in TZDs group compared with control (RR 2.96, 95% CI 1.22 to 7.20, I^2^ = 0%) (Fig. 6). We also did subgroup analyses by type of renal impairment, type of TZDs and type of control, but the subgroup differences had no statistical significance (Supplementary Table 3). No cohort study reported edema.

#### Cardiovascular events

Of the five trials (n = 233) reporting heart failure, one^17^ compared TZD versus active drugs (sulfonylureas), and the remaining four^16, 26, 27, 33^ compared TZDs versus placebo/no additional drugs. One trial^33^ used rosiglitazone as intervention and the other three trials used pioglitazone as intervention. Meta-analysis showed that TZDs-treatment did not increase the risk of heart failure (RR 0.64, 95% CI 0.15 to 2.66, I^2^ = 0%) (Table 2).

**Table 2:** Risk of cardiovascular events and mortality in patients with diabetes mellitus and renal impairment for the TZDs versus control groups.

Outcomes	No. of Study	Events/Total		RR (95%CI)	P Value	I^2^
		TZDs	Control			
Heart failure	5 RCTs	2/120	4/113	0.64 (0.15, 2.66)	0.54	0%
Angina	3 RCTs	2/84	1/84	1.45 (0.23, 8.95)	0.69	0%
MI	2 RCTs	0/45	0/46	—	—	—
Cardiovascular Mortality	1 RCT	0/26	1/26	0.33 (0.01, 7.82)	0.50	—
	1 cohort study	29/177	273/2050	1.23 (0.87, 1.75)	0.25	—
All-cause Mortality	5 RCTs	1/323	4/555	0.40 (0.08, 2.01)	0.27	0%
	2 cohort studies	74/407	714/2716	0.78 (0.38, 1.59)	0.62	85%

Three trials reported three angina events occurring in 168 patients. All these trials used pioglitazone as intervention. The pooling of those trials showed no statistically significant difference in the risk of angina between pioglitazone treatment and control (RR 1.45, 95% CI 0.23 to 8.95; I^2^ = 0%) (Table 2). Two trials^16, 33^ reported myocardial infarction, but no event occurred in each group. No study reported the data of stroke.

One trial^34^ (RR 0.33, 95% CI 0.01 to 7.82) and one cohort study^15^ (RR 1.23, 95% CI 0.87 to 1.75) reported cardiovascular mortality, both results showed that TZDs treatment may not increase the risk of cardiovascular mortality (Table 2).

#### All-Cause Mortality

Five trials involving 878 participants reported all-cause mortality, of which three trials^17, 23, 28^ compared TZDs versus active drugs (sufonylureas, DPP-4, metformin), two trials^33, 34^ compared TZDs versus placebo/no additional drugs. Meta-analysis showed that TZDs were not associated with increased risk of all-cause mortality (RR 0.40, 95% CI 0.08 to 2.01; I^2^ = 0%) (Table 2).

Meta-analysis of two cohort studies (n = 3,133) also showed that compared with control, TZDs did not increase the risk of all-cause mortality (RR 0.78, 95% CI 0.38 to 1.59; I^2^ = 85%) (Table 2).

We did sensitivity analyses using pooling methods and statistical models regarding heterogeneity, and the results (hypoglycemia, weight, risk of edema, HF, angina and all-cause mortality) were similar.

## Discussion

In this study, we found that TZDs may achieve improved glucose control relative to placebo in patients with diabetes and impaired renal functions. We did not observe difference in the glucose control effects between TZDs and other anti-diabetes medications. However, the effects of the two individual agents of TZDs on serum lipids may differ – subgroup analyses suggested that pioglitazone could elevate HDL and reduce TG and TC, but not with rosiglitazone. Our study also found that TZDs did not increase the risk of hypoglycemia. Compared with controls, TZDs significantly increased the risk of weight gain and edema, but their effects on heart failure, angina, myocardial infraction, cardiovascular mortality and all-cause mortality were uncertain.

Several previous studies supported our findings. A meta-analysis^37^ found that metformin, TZDs and sulfonylureas had similar hypoglycemic effect, which explained our findings that the changes of HbA1c and FPG had no statistical significance when compared with active drugs. The PROspective pioglitazone Clinical Trial in Macrovascular Events (PROactive) found that compared with placebo, pioglitazone reduced HbA1c, TG levels and increased HDL levels^38^. A meta-analysis including 23 RCTs, which compared pioglitazone or rosiglitazone against placebo in patients with type 2 diabetes, found that pioglitazone elevated HDL levels and reduced TG levels, but rosiglitazone increased LDL and TC levels^39^. Although TZDs exerted effects in reducing glucose, our meta-analysis indicated that TZDs played no role in the risk of hypoglycemia. Possible explanation behind this mechanism is the fact that TZDs are mainly metabolized by the liver. Indeed, current clinical practice guidelines recommendations state that TZDs can be used in patients with renal failure as TZDs do not increase the risk of hypoglycemia^2, 18^.

Although the adverse effects of weight gain and edema in TZDs-treated patients were established^11, 39–41^, the effects of TZDs on cardiovascular events and mortality remain to be further explored. On one hand, our findings were consistent with a few published meta-analyses that found treatment with TZDs did not increase the risk of MI^42, 43^, cardiovascular mortality, or all-cause mortality^44, 45^. The PROactive trial even showed that treatment with pioglitazone significantly reduced the risk of all-cause mortality, non-fatal MI and stroke^38^. These benefits may also exist in patients without diabetes^46^. The mechanism for the observed phenomenon is unclear. Since endothelial dysfunction is a strong predictor for future cardiovascular events in patients with coronary artery disease^47, 48^, one possible explanation is that TZDs may have benefit on endothelial function. TZDs, PPAR γ agonists, could exhibit anti-inflammatory properties^49, 50^ and increase NO release from endothlial cells^51^, which may produce vasodilatation and attenuate vascular damage. Moreover, pioglitazone treatment could increase the number and function of endothelial progenitor cells (EPCs). Increased levels of EPCs, which enhance angiogenesis, promote vascular repair, and improve endothelial function^52^, could reduce the risk of cardiovascular mortality in patients with coronary artery disease^48^. In patients with type 2 diabetes, pioglitazone showed an effect on ameliorating endothelial dysfunction, which was independent of its metabolic action^53, 54^. Indeed, a randomized, placebo-controlled, double-blind trial found that, after six months treatment, pioglitazone group had a significantly better coronary endothelial function compare to control^55^, further supporting the hypothesis.

However, a couple of other meta-analyses suggested that treatment with TZDs might increase risk of MI^8, 45^. The reasons of these seemingly conflicts may be due to the inclusion of different TZDs in those studies. Previous studies showed that pioglitazone may reduce ischemic disease and all-cause mortality in patients with type 2 diabetes^38^, while rosiglitazone may increase cardiovascular events especially MI^8, 9, 56^. The underlying mechanisms for the apparent differences in cardiovascular risk and mortality have not been clearly understood, but one possible explanation is that the two class of TZDs may have different effect on lipids, as mentioned above. And lipid abnormalities may cause endothelial cell toxicity and subsequently induce endothelial dysfunction. Moreover, pioglitazone has shown some potential benefit in preventing progression of atherosclerosis^57^, but rosiglitazone failed to show any potential benefit in this regard^58^.

Our study has several strengths. Firstly, to the best of our knowledge, this is the first systematic review and meta-analysis on the topic of efficacy and safety of TZDs treatment in patients with different degree of renal impairment. A published meta-analysis illustrated that TZDs significantly decreased urinary albumin and protein excretion in patients with diabetes, but they enrolled patients with normoalbuminuria or proteinuria^6^. Secondly, we systematically identified and included both randomized and non-randomized studies. Compared to previous reviews, we assessed both efficacy and safety outcomes of TZD-treated patients using quantitative methods. However, our findings should be interpreted cautiously due to some limitations. First of all, the risk of bias of most eligible studies were moderate to high. Secondly, due to limited number of the included studies, some subgroup analyses were not carried out. We could not analysis whether different type of TZDs have different effects on hypoglycemia, CV events and mortality. Thirdly, lack of universal standard to definite outcomes may add heterogeneity to this analyses.

In summary, this meta-analysis suggests TZDs treatment in diabetes patients with renal impairment may improve glucose control and serum lipid, but may increase the risk of weight gain and edema. However, the effects of TZDs on cardiovascular events and all-cause mortality were uncertain, mainly because of the limited sample sizes and inadequate power. More carefully designed, conducted, adequately powered studies (both RCTs and observational studies) are warranted to examine the effect on the long-term patient important outcomes.

## Methods

We followed the standards set by Preferred Reporting Items for Systematic reviews and Meta-Analyses (PRISMA)^59^ and the Meta-analysis Of Observational Studies in Epidemiology (MOOSE)^60^ in this systematic review.

### Eligibility criteria

We included randomized controlled trials (RCTs) that compared TZDs against placebo/no additional drugs or other hypoglycemic agents in patients with diabetes mellitus and renal impairment. Eligible studies should report at least one of the following outcomes: (1) glucose level: glycosylated hemoglobin (HbA1c), fasting plasma glucose (FPG), (2) serum lipids and lipoproteins: triacylglycerol (TG), total cholesterol (TC), low-density lipoprotein (LDL) and high-density lipoprotein (HDL), and (3) patient-important outcomes: hypoglycemia, weight, edema, cardiovascular events (heart failure (HF), angina pectoris, myocardial infarction (MI), stroke, cardiovascular mortality) and all-cause mortality. For patient-important outcomes, we also included cohort studies and case-control studies. Renal impairment was defined according to the presence or absence of kidney damage (abnormalities in pathological, urine, blood, or imaging test) and levels of kidney function. The degree of renal impairment was classified as non-ESRD (presence of kidney damage and/or decreased kidney function, not on dialysis), and ESRD (receiving dialysis or kidney transplantation).

### Literature search

A systematic literature search of PubMed, EMBASE, and Cochrane Central Register of Controlled Trials (CENTRAL) was performed from inception to 15 March 2016. We combined both MeSH and free words terms about “diabetes mellitus”, “chronic kidney disease” and “thiazolidinediones” for identifying relevant articles (Supplementary Text 1). We also screened ClinicalTrials.gov and reference lists of published reviews to identify additional relevant studies. Only studies published in English were included.

### Study screening and data collection

Two authors (WW and XZ) independently screened titles/abstracts and full-text articles to identify eligibility, assessed risk of bias, and collected data from each eligible study. We used standardized, pilot-tested forms, together with detailed instructions. For the included studies, we extracted data regarding study characteristics (study design, total number of patients, length of follow up and number of patients lost to follow up), baseline characteristics (gender, age, duration of diabetes, type of renal impairment, FPG, HbA1c), intervention and outcomes of interest. Disagreement was resolved through discussion or, if required, adjudication by a third author (JSWK).

### Risk of bias assessment

We assessed risk of bias of RCTs using a modified version of the Cochrane Collaboration’s tool for assessing risk of bias^61, 62^. The items included random sequence generation, allocation concealment, blinding of participants, caregivers, and assessors of outcomes, selective reporting, adequate follow up, and comparability.

We used the modified Newcastle-Ottawa Scale (NOS) for assessing the quality of cohort studies. We removed “representativeness of the exposed cohort” and “was follow-up long enough for outcomes to occur” as these items relate to applicability of results, and added “assessment of prognostic factors” and “similar co-interventions” to assess comparability between groups^63, 64^.

### Data synthesis and analysis

We analyzed RCTs and observational studies separately using risk ratios (RRs) for dichotomous outcomes and mean differences (MDs) for continuous outcomes with corresponding 95% confidence intervals (CIs) to compare difference between TZDs and control groups. We pooled RRs using the Mantel-Haenszel method, and MD using the inverse variance method. Statistical heterogeneity among studies was examined by the Chi-square test and quantified by the I^2^ statistic^65^. We explored sources of heterogeneity using the following subgroup analyses: type of renal impairment (non-ESRD vs. ESRD); type of TZDs (pioglitazone vs. rosiglitazone) and type of control (placebo/no additional drugs vs. active treatment). We carried out sensitivity analyses by using alternative pooling methods (Peto vs. Mantel-Haenszel method), and statistical models regarding heterogeneity (random-effects vs. fixed-effect). We also detected publication bias by visually examining symmetry of funnel plots and Egger’s tests.

## Electronic supplementary material

Efficacy and safety of thiazolidinediones in diabetes patients with renal impairment: a systematic review and meta-analysis
