# Supplementary material for: Efficacy and safety of thiazolidinediones in diabetes patients with renal impairment: a systematic review and meta-analysis
Source: Sci Rep. 2017 May 11;7:1717. doi: 10.1038/s41598-017-01965-0 (PMC5431943; doi:10.1038/s41598-017-01965-0)
Supplement: Supplementary file 1 — Efficacy and safety of thiazolidinediones in diabetes patients with renal impairment: a systematic review and meta-analysis [file 41598_2017_1965_MOESM1_ESM.pdf]

Efficacy and safety of thiazolidinediones in diabetes patients with renal impairment: a systematic review and meta-analysis

Short running title: Thiazolidinediones for diabetes patients with renal impairment

Authors

Wen Wang, MD, *Ph.D. candidate* [a], Xu Zhou, *Ph.D.*[a,d], Joey S.W. Kwong, *Ph.D.* [a,b], Ling Li, *Ph.D. candidate* [a,b], Youping Li, *M.D.*[a,b], Xin Sun, *Ph.D.* [a,b,c]

Supplementary text1 Search strategy

PubMed

1	"Diabetic Nephropathies" [MH]
2	"Kidney Diseases, Cystic"[MH]
3	Nephritis[MH]
4	"Renal Insufficiency, Chronic"[MH]
5	"Renal Dialysis"[MH]
6	Uremia[MH]
7	Renal Insufficiency"[MH]
8	chronic kidney[tw]
9	chronic renal[tw]
10	progressive kidney[tw]
11	diabetic kidney[tw]
12	diabetic renal[tw]
13	kidney disease*[tw]
14	kidney impair*[tw]
15	kidney failur*[tw]
16	kidney function*
17	kidney insufficiency[tw]
18	kidney disorder*[tw]
19	kidney dysfunction[tw]
20	renal disease*[tw]
21	renal impair*[tw]
22	renal failur*[tw]
23	renal function*[tw]
24	renal insufficiency[tw]
25	renal disorder*[tw]
26	renal dysfunction[tw]
27	glomerular disease*[tw]
28	glomerular disorder*[tw]
29	glomerular dysfunction[tw]
30	kidney fibrosis[tw]
31	renal fibrosis[tw]
32	glomerular fibrosis[tw]
33	tubulointerstitial fibrosis[tw]
34	endstage kidney disease*[tw]
35	end stage kidney disease*[tw]
36	dialysis*[tw]
37	hemodia*[tw]
38	haemodia*[tw]
39	hemofiltration*[tw]
40	haemofiltration*[tw]
41	hemodiafiltration*[tw]
42	haemodiafiltration*[tw]
43	tenckhoff[tw]
44	proteinuri*[tw]
45	microalbuminuri*[tw]
46	macroalbuminuri*[tw]
47	albuminuri*[tw]
48	hypoalbuminemi*[tw]
49	hypoalbuminaemi*[tw]
50	glomerulopath*[tw]
51	alport[tw]
52	denys-drash[tw]
53	uremi*[tw]
54	uraemi*[tw]
55	multicystic kidney[tw]
56	polycystic kidney[tw]
57	cystic kidney[tw]
58	nephritis*[tw]
59	nephrop*[tw]
60	nephrotic syndrome[tw]
61	1 OR 2 OR 3 OR 4 OR 5 OR 6 OR 7 OR 8 OR 9 OR 10 OR 11 OR 12 OR 13 OR 14 OR 15 OR 16 OR17 O18 OR 19 OR 20 OR 21 OR 22 OR 23 OR 24 OR 25

62 OR 26 OR 27 OR 28 OR 29 OR 30 OR 31 OR 32 OR 33 OR 34 OR 35 OR 36 OR 37 OR 38 OR 39 OR 40 OR 41 OR 42 OR 43 OR 44 OR 45 OR 46 OR 47 OR 48 OR 49 OR 50 OR 51 OR 52 OR 53 OR 54 OR 55 OR 56 OR 57 OR 58 OR 59 OR 60

63 "Hypoglycemic Agents"[mh]

64 Thiazolidinediones[mh]

65 Thiazolidinedione*[tw]

66 rosiglitazone[tw]

67 pioglitazone[tw]

68 peroxisome proliferator-activated receptor agonist*[tw]

69 ppar agonist*.tw.

70 62 OR 63 OR 64 OR 65 OR 66 OR 67 OR 68

71 Randomized controlled trial[pt]

72 Controlled clinical trial[pt]

73 comparative study[pt]

74 "Clinical Trials as Topic"[mh]

75 "case-control studies"[mh]

76 "Cohort Studies"[mh]

77 "Longitudinal Studies"[mh]

78 "retrospective studies"[mh]

79 "Follow-Up Studies"[mh]

80 "prospective studies"[mh]

81 "Registries"[mh]

82 Random*[tiab]

83 nonrandom*[tiab]

84 non random*[tiab]

85 trial[tiab]

86 trials[tiab]

87 longitudinal*[tiab]

88 prospective*[tiab]

89 retrospective*[tiab]

90 incidence stud*[tiab]

91 concurrent stud*[tiab]

92 group*[tiab]

93 database*[tiab]

94 population*[tiab]

95 registr*[tiab]

96 70 OR 71 OR 72 OR 73 OR 74 OR 75 OR 76 OR 77 OR 78 OR 79 OR 80 OR 81 OR 82 OR 83 OR 84 OR 85 OR 86 OR 87 OR 88 OR 89 OR 90 OR 91 OR 92 OR 93 OR 94

97 English[la]

98 61 AND 69 AND 95 AND 96

99 "Review Literature as Topic"[MH]

100 "Meta-Analysis as Topic"[MH]

101 "Meta-Analysis"[pt]

102 review[pt]

103 comment[pt]

104 letter[pt]

105 editorial[pt]

106 meta analy*[tw]

107 Metaanaly*[tw]

108 systematic review*[tw]

109 98 OR 99 OR 100 OR 101 OR 102 OR 103 OR 104 OR 105 OR 106 OR 107

110 97 NOT 108

111 animal[mh]

112 humans[mh]

113 110 NOT 111

114 109 NOT 112

Embase and CENTRAL (via OVID)

1 exp chronic kidney disease/
2 exp chronic kidney failure/
3 chronic kidney.tw.
4 chronic renal.tw.
5 progressive kidney.tw.
6 diabetic kidney.tw.
7 diabetic renal.tw.
8 kidney disease*.tw.
9 kidney impair*.tw.
10 kidney failur*.tw.
11 kidney function*.tw.
12 kidney insufficiency.tw.
13 kidney disorder*.tw.
14 kidney dysfunction.tw.
15 renal disease*.tw.
16 renal impair*.tw.
17 renal failur*.tw.
18 renal function*.tw.
19 renal insufficiency.tw.
20 renal disorder*.tw.
21 renal dysfunction.tw.
22 glomerular disease*.tw.
23 glomerular disorder*.tw.
24 glomerular dysfunction.tw.
25 kidney fibrosis.tw.
26 renal fibrosis.tw.
27 glomerular fibrosis.tw.
28 tubulointerstitial fibrosis.tw.
29 endstage kidney disease*.tw.
30 end stage kidney disease*.tw.
31 dialysis*.tw.
32 hemodia*.tw.
33 haemodia*.tw.
34 hemofiltration*.tw.
35 haemofiltration*.tw.
36 hemodiafiltration*.tw.
37 haemodiafiltration*.tw.
38 tenckhoff.tw.
39 proteinuri*.tw.
40 microalbuminuri*.tw.
41 macroalbuminuri*.tw.
42 albuminuri*.tw.
43 hypoalbuminemi*.tw.
44 hypoalbuminaemi*.tw.
45 glomerulopath*.tw.
46 alport.tw.
47 denys-drash.tw.
48 uremi*.tw.
49 uraemi*.tw.
50 multicystic kidney.tw.
51 polycystic kidney.tw.
52 cystic kidney.tw.
53 nephritis*.tw.
54 nephrop*.tw.
55 nephrotic syndrome.tw.
56 1 OR 2 OR 3 OR 4 OR 5 OR 6 OR 7 OR 8 OR 9 OR 10 OR 11 OR 12 OR 13 OR 14 OR 15 OR 16 OR17 O18 OR 19 OR 20 OR 21 OR 22 OR 23 OR 24 OR 25 OR 26 OR 27 OR 28 OR 29 OR 30 OR 31 OR 32 OR 33 OR 34 OR 35 OR 36 OR 37 OR 38 OR 39 OR 40 OR 41 OR 42 OR 43 OR 44 OR 45 OR 46 OR 47 OR 48 OR 49 OR 50 OR 51 OR 52 OR 53 OR 54 OR 55
57 exp antidiabetic agent/
58 Thiazolidinedione*.tw.
59 rosiglitazone.tw.
60 pioglitazone.tw.
61 peroxisome proliferator-activated receptor agonist*.tw.
62 ppar agonist*.tw.
63 57 OR 58 OR 59 OR 60 OR 61 OR 62
64 clinical trials as topic/
65 controlled clinical trials as topic/
66 randomized controlled trials as topic/
67 case-control studies/
68 cohort studies/
69 longitudinal studies/
70 retrospective studies/
71 follow-up studies/
72 prospective studies/
73 registries/
74 random*.ti,ab nonrandom*.ti,ab.

75 non random*.ti,ab.
76 trial.ti,ab.
77 trials.ti,ab.
78 cohort*.ti,ab.
79 case control*.ti,ab.
80 case control*.ti,ab.
81 follow up*.ti,ab.
82 followup*.ti,ab.
83 longitudinal*.ti,ab.
84 prospective*.ti,ab.
85 retrospective*.ti,ab.
86 group*.ti,ab.
87 database*.ti,ab.
88 population*.ti,ab.
89 registr*.ti,ab.
90 64 OR 65 OR 66 OR 67 OR 68 OR 69 OR 70 OR 71 OR 72 OR 73 OR 74 OR 75 OR 76 OR 77 OR 78 OR 79 OR 80 OR 81 OR 82 OR 83 OR 84 OR 85 OR 86 OR 87 OR 88 OR 89
91 limit 90 to english language
92 limit 91 to human [Limit not valid in CANTRAL; records were retained]

Supplementary Table 1 Risk of bias of included studies

Risk of bias in randomized controlled trials

Of the 19 RCTs, nine (47.4%) reported adequate random sequence generation, seven (36.8%) blinded patients and six (31.6%) blinded care givers and patients. A total of 14 (73.7%) trials followed up more than 90% patients, but only three adequately compared prognostic factors. In conclusion, these 19 RCTs were at moderate to high risk of bias, of which ten (52.6%) trials were at high risk, the remaining 9 (47.4%) trials were at moderate risk (Table 1).

Table 1 *Risk of bias of randomized controlled trials*

**Study**	**Adequate randomization sequence generation**	**Adequate allocation concealment**	**Adequate blinding of patients**	**Adequate blinding of care givers**	**Blinding of outcome assessor**	**Free from selective reporting**	**Adequate follow up**	**Adequate comparability of prognostic factors**	**Qualitative of risk of bias**
Abe 2007 [19]	Definitely yes	Probably no	Definitely no	Definitely no	Definitely no	Definitely yes	Definitely yes	Probably no	High risk
Abe 2008 [20]	Probably yes	Probably no	Definitely no	Definitely no	Definitely no	Definitely yes	Definitely yes	Probably no	High risk
Abe 2010 [16]	Definitely yes	Probably no	Definitely no	Definitely no	Definitely no	Definitely yes	Definitely yes	Probably no	High risk
Agarwal 2005 [17]	Definitely yes	Definitely yes	Definitely no	Definitely no	Definitely no	Definitely yes	Definitely yes	Definitely yes	Moderate risk
Agrawal 2003 [21]	Probably yes	Probably yes	Definitely yes	Definitely yes	Probably yes	Definitely yes	Definitely yes	Probably no	Moderate risk
Arashnia 2015 [22]	Probably yes	Probably yes	Definitely yes	Definitely yes	Probably yes	Definitely yes	Definitely yes	Probably no	Moderate risk
Banerji 2010 [23]	Definitely yes	Definitely yes	Definitely no	Definitely no	Definitely no	Definitely yes	Definitely yes	Probably no	High risk
Chan 2011 [24]	Definitely yes	Probably yes	Definitely yes	Definitely yes	Probably yes	Definitely yes	Definitely yes	Probably no	Moderate risk
Galle 2012 [25]	Probably yes	Probably yes	Definitely yes	Definitely yes	Probably yes	Definitely yes	Probably no	Definitely yes	Moderate risk
Jin 2007 [26]	Definitely yes	Probably yes	Probably no	Probably no	Probably no	Definitely yes	Definitely yes	Probably no	Moderate risk
Katavetin 2006 [27]	Definitely yes	Definitely yes	Definitely yes	Definitely no	Probably no	Definitely yes	Probably yes	Probably no	Moderate risk
Morikawa 2011 [28]	Probably yes	Probably yes	Definitely no	Definitely no	Definitely no	Definitely yes	Definitely yes	Definitely yes	Moderate risk
Nakamura 2000 [29]	Probably yes	Probably yes	Probably no	Probably no	Probably no	Definitely yes	Probably yes	Probably no	High risk
Nakamura 2001 [30]	Probably yes	Probably yes	Definitely yes	Definitely yes	Probably no	Definitely yes	Probably yes	Probably no	Moderate risk
Nakamura 2004 [31]	Definitely yes	Definitely yes	Probably no	Probably no	Probably no	Definitely yes	Definitely yes	Probably no	High risk

Nakamura 2006 [32]	Probably yes	Definitely yes	Probably no	Probably no	Probably no	Definitely yes	Definitely yes	Probably no	High risk
Pistrosch 2012 [33]	Probably yes	Probably yes	Definitely yes	Definitely yes	Probably yes	Definitely yes	Probably yes	Probably no	Moderate risk
Wong 2005 [34]	Definitely yes	Definitely yes	Definitely no	Definitely no	Definitely no	Definitely yes	Definitely yes	Probably no	High risk
Yanagawa 2004 [35]	Probably yes	Probably no	Probably no	Probably no	Probably no	Definitely yes	Definitely yes	Probably no	High risk

Risk of bias in cohort studies

Of the three observational studies, all studies selected exposure and control from same source, and all studies had confident in ascertaining exposure and control. Two studies conduct well adjustment for prognostic factors between exposure and control cohort. In conclusion, one study was at low risk of bias, one were at moderate risk and another one was at high risk (Table 2).

Table 2 *Risk of bias of cohort studies*

**Author**	**Selection from same populati on**	**Ascertain ment of exposure**	**Ascertain ment of control**	**Outcome of interest was not present at baseline**	**Adequate comparability of prognostic factors**	**Assessmen t of prognostic factors**	**Ascertain ment of outcomes**	**Adequate follow up**	**Similar co-interventio ns**	**Qualitativ e of risk of bias**
Brunelli 2009 [14]	Definitel y yes	Definitely yes Drug prescription in the routine clinical practice	Definitely yes Drug prescription in the routine clinical practice	Definitel y yes	Definitely yes Cox proportional hazards models adjusted age, race, sex, body mass index, facility standardized mortality ratio	Definitely yes From electronic health record	Definitely yes From electronic health record	Probably no Retrospecti ve cohort	Probably no	Moderate risk
Chen YH 2015 [36]	Definitel y yes	Definitely yes Drug prescription in the routine clinical practice	Definitely yes Drug prescription in the routine clinical practice	Definitel y yes	Definitely no	Definitely yes From electronic health record	Definitely yes From electronic health record	Probably no Retrospecti ve cohort	Probably no	High risk
Ramirez2 009 [15]	Definitel y yes	Definitely yes From a prospective cohort	Definitely yes From a prospective cohort	Definitel y yes	Definitely yes Adjustments included the two TZD types and insulin; age, gender, race BMI, years with ESRD, comorbid conditions, hemoglobin, serum glucose, total cholesterol concentration	Probably yes	Definitely yes From a prospective cohort	Probably yes	Probably yes	Low risk

Supplementary Table 3 Subgroup analyses of HbA1c, FPG, serum lipids and edema

Table 3 Subgroup analyses of HbA1c, FPG, serum lipids and edema

Outcomes	No. of Studies (patients)	Effect estimate (95% CI)	P value of test for overall effect	I^2	P value of interaction
**HbA1c**					
**Different degree of RI**					
Non-ESRD	10 (623)	-0.57 (-1.05, -0.08)	0.02	73%	0.68
ESRD	5 (222)	-0.69 (-1.04, -0.35)	<0.0001	55%	
**Different TZDs**					
Pioglitazone	12 (476)	-0.64 (-0.97, -0.31)	0.0001	62%	0.97
Rosiglitazone	3 (369)	-0.62 (-1.51, 0.27)	0.17	83%	
**Different control**					
Placebo/no additional drugs	9 (607)	-0.90 (-1.24, -0.56)	<0.00001	73%	0.003
Active drugs	6 (238)	-0.16 (-0.50, 0.18)	0.36	0%	
**FPG**					
**Different degree of RI**					
Non-ESRD	5 (467)	-25.17 (-54.66, 4.31)	0.09	95%	0.97
ESRD	5 (228)	-20.66 (-36.77, -4.56)	0.01	32%	
**Different TZDs**					
Pioglitazone	8 (336)	-27.17 (-50.18, -4.16)	0.02	84%	0.91
Rosiglitazone	2 (359)	-24.23 (-68.33, 19.87)	0.28	97%	
**Different control**					
Placebo/no additional drugs	8 (615)	-32.26 (-53.13, -11.39)	0.002	90%	0.008
Active drugs	2 (80)	3.94 (-12.96, 20.84)	0.65	0%	
**TG**					
**Different degree of RI**					
Non-ESRD	5 (213)	-21.41 (-55.71, 12.88)	0.13	29%	0.83
ESRD	6 (264)	-16.84 (-38.89, 5.21)	0.22	75%	
**Different TZDs**					
Pioglitazone	8 (327)	-26.38 (-40.56, -12.19)	0.0003	25%	0.05
Rosiglitazone	3 (150)	31.81 (-24.73, 88.35)	0.08	61%	
**Different control**					
Placebo/no additional drugs	9 (402)	-15.57 (-38.83, 7.70)	0.19	73%	0.84
Active drugs	2 (75)	0.35 (-152.80, 153.50)	1.00	0%	
**TC**					
**Different degree of RI**					
Non-ESRD	7 (311)	-3.07 (-12.89, 6.76)	0.54	29%	0.26
ESRD	5 (206)	8.09 (-8.61, 24.79)	0.34	44%	
**Different TZDs**					
Pioglitazone	9 (367)	-7.00 (-13.77, -0.23)	0.04	0%	0.006
Rosiglitazone	3 (150)	13.51 (0.48, 26.54)	0.04	0%	
**Different control**					
Placebo/no additional drugs	9 (402)	3.75 (-6.75, 14.26)	0.48	46%	0.17

Active drugs	3 (115)	-8.32 (-21.76, 5.11)	0.22	0%	
**LDL**					
**Different degree of RI**					
Non-ESRD	2 (110)	9.48 (-1.35, 20.31)	0.09	0%	0.48
ESRD	3 (130)	1.95 (-15.86, 19.76)	0.83	53%	
**Different TZDs**					
Pioglitazone	3 (118)	8.30 (-12.82, 29.41)	0.44	73%	0.77
Rosiglitazone	2 (122)	4.66 (-6.61, 15.94)	0.42	0%	
**Different control**					
Placebo/no additional drugs	4 (200)	2.76 (-9.96, 15.47)	0.67	55%	0.39
Active drugs	1 (40)	12.00 (-4.93, 28.93)	0.16	-	
**HDL**					
**Different degree of RI**					
Non-ESRD	4 (185)	3.12 (-0.52, 6.77)	0.09	6%	0.74
ESRD	6 (264)	4.02 (0.18, 7.87)	0.04	59%	
**Different TZDs**					
Pioglitazone	8 (327)	4.84 (2.50, 7.18)	<0.0001	22%	0.01
Rosiglitazone	2 (122)	-1.92 (-6.66, 2.82)	0.43	0%	
**Different control**					
Placebo/no additional drugs	8 (374)	3.54 (0.47, 6.60)	0.02	47%	0.83
Active drugs	2 (75)	4.34 (-2.51, 11.20)	0.21	56%	
**Edema**					
**Different degree of RI**					
Non-ESRD	5 (1169)	2.94 (1.11, 7.76)	0.03	0%	0.98
ESRD	2 (103)	3.05 (0.33, 28.32)	0.33	0%	
**Different TZDs**					
Pioglitazone	4 (208)	1.67 (0.36, 7.75)	0.51	0%	0.67
Rosiglitazone	2 (371)	3.83 (0.64, 23.08)	0.14	0%	
TZDs	1 (693)	4.03 (1.02, 15.95)	0.05	-	
**Different control**					
Placebo/no additional drugs	4 (474)	3.50 (0.86, 14.18)	0.08	0%	0.64
Active drugs	3 (798)	2.03 (0.34, 12.15)	0.44	43%	

Supplement Figure 1 Risk of hypoglycemia in diabetes patients with renal impairment for the TZDs versus control groups from RCTs

	TZDs		Control			Risk Ratio
Study or Subgroup	Events	Total	Events	Total	Weight	M-H, Random, 95% CI
Abe 2010	0	31	0	32		Not estimable
Agarwal 2006	2	21	1	21	12.3%	2.00 [0.20, 20.41]
Agrawal 2003	7	145	4	156	45.5%	1.88 [0.56, 6.30]
Banerji 2010	0	230	5	463	7.9%	0.18 [0.01, 3.29]
Galle 2012	2	20	2	19	19.2%	0.95 [0.15, 6.08]
Katavetin 2006	4	24	1	16	15.1%	2.67 [0.33, 21.73]
**Total (95% CI)**		**471**		**707**	**100.0%**	**1.46 [0.65, 3.29]**
Total events	15		13			

Heterogeneity: Tau² = 0.00; Chi² = 2.84, df = 4 (P = 0.58); I² = 0%
Test for overall effect: Z = 0.90 (P = 0.37)

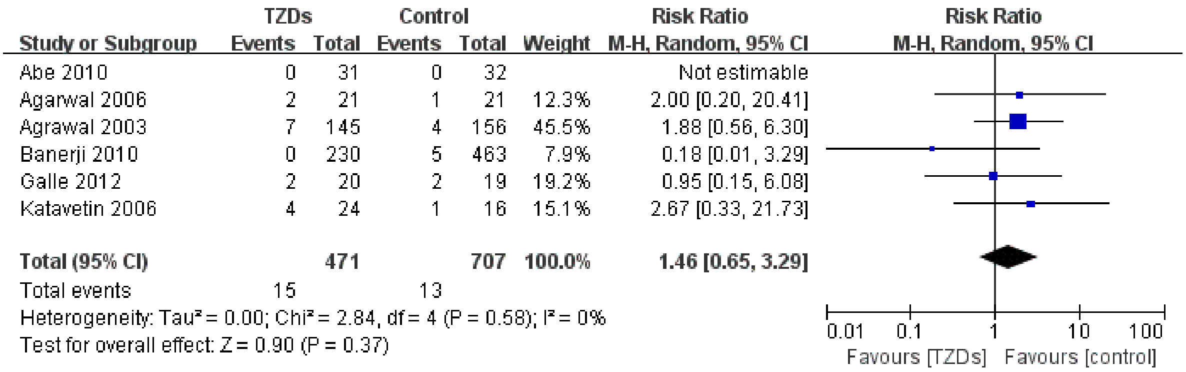

Fig 1 Risk of hypoglycemia in patients with diabetes mellitus and renal impairment for the TZDs versus control groups from RCTs
